# Antibody response to inactivated COVID‐19 vaccine in patients with type 2 diabetes mellitus after the booster immunization

**DOI:** 10.1111/1753-0407.13448

**Published:** 2023-07-30

**Authors:** Haolong Li, Yao Wang, Xiaomeng Li, Siyu Wang, Xinxin Feng, Xinhua Xiao, Yongzhe Li

**Affiliations:** ^1^ Department of Clinical Laboratory, State Key Laboratory of Complex Severe and Rare Diseases Peking Union Medical College Hospital, Chinese Academy of Medical Science and Peking Union Medical College Beijing China; ^2^ Department of Clinical Laboratory Peking University People's Hospital Beijing China; ^3^ Department of Endocrinology Peking Union Medical College Hospital Beijing China

**Keywords:** antibodies, immune response, SARS‐CoV‐2, type 2 diabetes mellitus, vaccination, 抗体, 免疫反应, SARS‐CoV‐2, 疫苗接种, 2型糖尿病

## Abstract

**Background:**

The immunogenicity of booster inactivated COVID‐19 vaccines in patients with type 2 diabetes mellitus (T2DM) has remained unclear. Our study aims to investigate the antibody response to inactivated COVID‐19 vaccine following booster vaccination in patients with T2DM.

**Methods:**

A total of 201 patients with T2DM and 102 healthy controls (HCs) were enrolled. The levels of anti‐SARS‐CoV‐2 total antibodies, anti‐receptor‐binding domain (RBD)‐specific IgG, neutralizing antibody (NAb) toward SARS‐CoV‐2 wild type (WT), and NAb toward SARS‐CoV‐2 Omicron BA.4/5 subvariant were measured to evaluate the vaccine‐induced immunological responses.

**Results:**

The titers of anti‐RBD‐specific IgG (*p* = 0.018) and inhibition rates of NAb toward WT (*p* = 0.007) were significantly decreased in patients with T2DM compared to HCs after booster vaccination for more than 6 months. Both HCs and patients with T2DM showed poor resistance against BA.4/5 due to the detected inhibition rates being lower than the positive threshold. The levels of anti‐RBD‐specific IgG were positively associated with the proportions of CD3^+^CD4^−^CD8^−^ T cells (*p* = 0.045), and patients with T2DM who had anti‐RBD‐specific IgG positivity showed higher proportions of CD3^+^CD4^−^CD8^−^ T cells compared to those negative (*p* = 0.005).

**Conclusions:**

Patients with T2DM showed impaired antibody responses after booster vaccination for more than 6 months. Decreased anti‐BA.4/5 responses give rise to the possibility of breakthrough infections for both patients with T2DM and HCs.

## Graphical abstract

**Highlights**

- Similar antibody response to COVID‐19 inactivated vaccine between healthy controls (HCs) and patients with type 2 diabetes mellitus (T2DM) after a booster shot vaccine within 6 months.
- Patients with T2DM have reduced antibody response after receiving the third dose of the COVID‐19 vaccine for more than 6 months.
- Both HCs and T2DM showed poor resistance against BA.4/5 due to the detected inhibition rates being lower than the positive threshold.

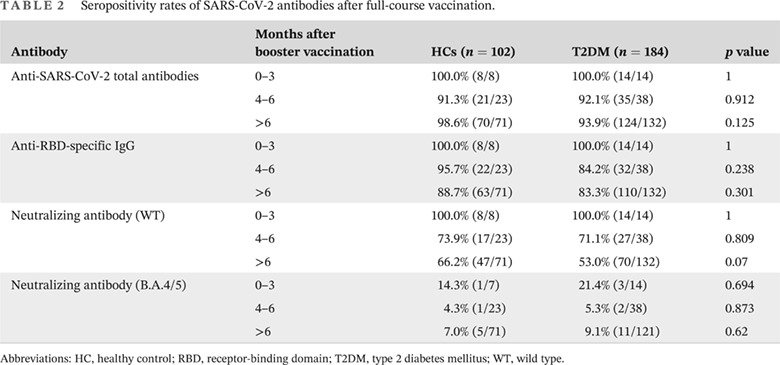

## 摘要

背景:目前尚不清楚2型糖尿病(T2DM)患者接种增强剂次灭活新型冠状病毒病(COVID‐19)疫苗后的免疫原性。本研究旨在调查T2DM患者在接种增强剂次灭活COVID‐19疫苗后的抗体反应。

方法:共纳入201例T2DM患者和102名健康对照(HCs)。测量抗严重急性呼吸综合症冠状病毒2(SARS‐CoV‐2)总抗体、针对受体结合结构域(RBD)特异性IgG、针对SARS‐CoV‐2野生型(WT)的中和抗体(NAb)以及针对SARS‐CoV‐2Omicron BA.4/5亚变体的NAb水平, 以评估疫苗接种诱导的免疫学反应。

结果:与HCs相比, T2DM患者在接种增强剂次疫苗后超过6个月的抗RBD特异性IgG滴度(P = 0.018)和针对WT的NAb抑制率(P = 0.007)显著降低。HCs和T2DM均表现出对BA.4/5的抵抗力较差, 因为检测到的抑制率低于阳性阈值。抗RBD特异性IgG水平与CD3+CD4‐CD8‐ T细胞的比例呈正相关(P = 0.045), 与阴性相比, T2DM患者的抗RBD特异性IgG阳性显示出更高比例的CD3+CD4‐CD8‐ T细胞(P = 0.005)。

结论:T2DM患者在接种增强剂次疫苗后超过6个月表现出抗体反应受损。针对BA.4/5的反应减弱使得T2DM患者和HCs都可能出现突破感染的可能性。

## 1 INTRODUCTION

The COVID‐19 pandemic caused by SARS‐CoV‐2 has persisted worldwide for more than 3 years. Patients with type 2 diabetes mellitus (T2DM) are at higher risk for SARS‐CoV‐2 infection and have a worse prognosis for COVID‐19 than healthy individuals.^1^ Currently, there are about more than 158 million patients with T2DM in China according to the latest epidemiological surveys,^2^ and the prevalence of diabetes was higher in the elderly population.^2^

The COVID‐19 vaccine is an effective means to protect against SARS‐CoV‐2 infection and to reduce the severity and mortality rates.^3^ Several studies have investigated the immunogenicity of the COVID‐19 vaccine in patients with T2DM. Ali et al. observed that patients with T2DM had lower levels of SARS‐CoV‐2 IgG and neutralizing antibodies (NAbs) compared to those without T2DM after receiving two doses of BNT162b2 mRNA vaccines.^4^ Sourij et al. reported that the titers of anti‐SARS‐CoV‐2 spike protein antibody were decreased in patients with T2DM compared with the healthy controls (HCs) after receiving the second dose of BioNTech‐Pfizer/Moderna mRNA‐based vaccines.^5^ However, Lee et al. found that both the levels of anti‐receptor‐binding domain (RBD) IgG antibody and NAb did not show significant differences between patients with T2DM and HCs after finishing full‐course vaccination of BNT162b2 mRNA vaccines.^6^ Therefore, the effectiveness of the COVID‐19 vaccine in patients with T2DM remains controversial. Along with the rapid emergence and spread of SARS‐CoV‐2 variants of concern (VOCs), the effectiveness of the COVID‐19 vaccine was decreased due to VOCs escaping from NAbs and/or cell‐mediated immunity.^7^ To date, the Omicron lineage BA.4/5 has spread throughout Asia,^8^ exhibiting higher infectivity and greater immune escape than the original SARS‐CoV‐2 strain of early 2020.^9^ Furthermore, the third dose of mRNA‐1273 vaccination showed poor vaccine effectiveness.^10^

In China, the inactivated vaccine (BBIBP‐CorV or CoronaVac) is the most used type of COVID‐19 vaccine. However, the data on the immunogenicity of inactivated COVID‐19 vaccines in patients with T2DM remained unclear, especially in those who receive booster inactivated vaccinations. The antibody responses to Omicron subvariants after receiving the third dose of COVID‐19 vaccines need to be identified in patients with T2DM. Therefore, this study aims to investigate the humoral immune response to SARS‐CoV‐2 and its VOCs (BA.4/5) in patients with T2DM following a third dose of inactivated COVID‐19 vaccine.

## 2 MATERIALS AND METHODS

### 2.1 Participants

Two hundred and one patients with T2DM and 102 HCs were enrolled in this cross‐sectional observational study between May 16, 2022, and September 13, 2022, at the Peking Union Medical College Hospital (PUMCH) which is located in Beijing, China. The inclusion criteria for T2DM were as follows: (1) Patients diagnosed with T2DM according to World Health Organization criteria and (2) patients who received at least the two‐dose regimen of inactivated vaccine (BBIBP‐CorV/CoronaVac) after the definite diagnosis of T2DM. For all individuals, the following exclusion criteria applied: (1) a history of SARS‐CoV‐2 infection; (2) positive reverse transcription polymerase chain reaction (PCR) results for SARS‐CoV‐2 on naso‐oropharyngeal swabs; (3) COVID‐19 symptoms such as fever, cough, or fatigue when sampling; and (4) age < 18. This study was approved by the institutional review board of PUMCH (K1965‐K22C0433). Written informed consent was obtained from all participants.

### 2.2 Data collection

Electronic medical records of patients with T2DM were collected including age, sex, body mass index (BMI), and comorbidities. Vaccination information of all participants was retrieved from the State Council client applet when they visited the PUMCH. A total of 184 patients with T2DM were categorized into three subgroups according to inoculation dose and duration at the time of sampling, including those with booster vaccination of the third dose after 0–3 months (*n* = 14), third dose after 4–6 months (*n* = 38), and third dose after more than 6 months (*n* = 132). HCs were also matched with the last vaccination period. Moreover, 17 patients with T2DM (*n* = 17) with full‐course vaccination were also enrolled. Fasting EDTA plasma was collected from each participant in the morning after 10 h of fasting and stored at −80°C. Their plasma sample was collected for the detection of anti‐SARS‐CoV‐2 total antibodies, anti‐RBD‐specific IgG antibody, NAb toward SARS‐CoV‐2 wild type (WT), and NAb toward the SARS‐CoV‐2 Omicron BA.4/5 subvariant.

### 2.3 Laboratory analysis

The concentration of fasting plasma glucose (FPG) was measured on the AU5800 automatic biochemical analyzer (Beckman Coulter, Brea, California). Glycosylated hemoglobin (HbA1c) was measured by ion‐exchange high‐performance liquid chromatography (HPLC; Bio‐Rad, Hercules, California). Lymphocyte immunophenotyping was performed by flow cytometry (Epics XL flow cytometry; Beckman Coulter, Brea, California). Specific monoclonal antibodies against CD3, CD4, CD8, CD19, CD16, and CD56 were used to identify lymphocyte subsets.

### 2.4 SARS‐CoV‐2 antibody test

The total antibodies against SARS‐CoV‐2 (anti‐SARS‐CoV‐2 total antibodies) were determined by double‐antigen sandwich enzyme‐linked immunosorbent assay (ELISA) using the total antibodies against SASR‐CoV‐2 detection kit (Wantai Biological Pharmacy Enterprise, Beijing, China). NAbs toward SARS‐CoV‐2 WT and the Omicron BA.4/5 subvariant were determined by competitive ELISA using the SARS‐CoV‐2 surrogate virus neutralization test (sVNT) assay (Genscript, Nanjing, China). The SARS‐CoV‐2 antibody against the spike protein RBD (anti‐RBD‐specific IgG) was detected by using a capture sandwich ELISA detection kit (PROPRIUM, Hangzhou, China). The details have been described in our previous study.^11^

### 2.5 Statistical analysis

The data are reported as mean ± SD and median (interquartile range, IQR) for continuous variables or number (percentage) for categorical variables. Continuous variables were compared using Student's *t*‐test or the Mann–Whitney *U* test. Categorical variables were compared using the chi‐square test or Fisher's exact test. One‐way analysis of variance (ANOVA) and the Kruskal–Wallis test were used to compare the results of multiple groups. Correlations of SARS‐CoV‐2 antibodies were calculated using Pearson's correlation analysis or Spearman's correlation analysis with a two‐tailed *p* value. *p* values <0.05 were considered statistically significant. The data were analyzed using IBM SPSS version 23.0 software. Hiplot (https://hiplot.org) was used to visualize the data.

## 3 RESULTS

### 3.1 Demographics and clinical characteristics of participants

A total of 184 patients with T2DM and 102 HCs received three doses of the COVID‐19 vaccine in this study; their demographic data and characteristics are shown in Table 1. In addition, detailed treatment information is presented in Table S1, and no participants received glucocorticoids, immunosuppressants, and other treatments that impair immune function. The age and gender were comparable between patients with T2DM and HCs, while patients with T2DM had increased levels of FPG (7.6 vs. 5.4, *p* < 0.001) and higher proportions of HbA1c (7.1 vs. 5.6, *p* < 0.001) than HCs. The median time after the third vaccination was 216 days (IQR 167.0–253.0 days) and 219 days (IQR 165.0–251.0 days) for the patients with T2DM and HCs, respectively (*p* = 0.537). Moreover, the characteristics of participants at 0–3, 4–6, and more than 6 months after boost vaccination are shown in Table S2. In addition, 17 patients with T2DM who received two doses of the COVID‐19 vaccine were also enrolled, and their characteristics are shown in Table S3.

**TABLE 1:** Characteristics of HCs and patients with T2DM who completed COVID‐19 booster vaccination.

Variables	HCs (*n* = 102)	T2DM (*n* = 184)	*p* value
Age (years)	64 (61–67)	66 (61–70)	0.061
<60, *n* (%)	10 (9.8%)	28 (15.2%)	0.196
≥60, *n* (%)	92 (90.2%)	156 (84.8%)	
Gender (male, *n* [%])	56 (54.9%)	102 (55.4%)	0.931
BMI (kg/m^2^)	24.24 (22.95–26.54)	25.30 (23.53–26.95)	0.065
<24, *n* (%)	45 (46.4%)	52 (30.4%)	0.031
24–28, *n* (%)	39 (40.2%)	86 (50.3%)	
≥28, *n* (%)	13 (13.4%)	33 (19.3%)	
FPG (mmol/L)	5.4 (5.1–5.7)	7.6 (6.8–8.8)	<0.001
HbA1c (%)	5.6 (5.4–5.8)	7.1 (6.6–7.8)	<0.001
CD3^+^CD4^+^ T cells (% of lymphocytes)	36.86 (31.63–44.55)	37.71 (32.74–44.78)	0.728
CD3^+^CD8^+^ T cells (% of lymphocytes)	22.83 (18.24–30.34)	23.51 (17.95–29.35)	0.992
CD3^+^CD4^−^CD8^−^ T cells (% of lymphocytes)	3.63 (2.62–5.78)	3.40 (2.27–4.86)	0.059
NK cells (% of lymphocytes)	17.22 (11.66–26.24)	17.22 (11.73–24.15)	0.808
B cells (% of lymphocytes)	9.88 (7.60–12.54)	10.94 (7.76–13.74)	0.083
Comorbidities			
Hypertension	0	114 (62.0%)	NA
Hyperlipemia	0	138 (75.0%)	NA
Chronic respiratory disease	0	21 (11.4%)	NA
Cardiovascular and cerebrovascular diseases	0	99 (53.8%)	NA
Liver diseases	0	20 (10.9%)	NA
Kidney diseases	0	18 (9.8%)	NA
Autoimmune diseases	0	2 (1.1%)	NA
Cancer	0	6 (3.3%)	NA
Period of the third vaccination at the time of sampling (day)	219 (165–251)	216 (167–253)	0.537

Abbreviations: BMI, body mass index; FPG, fasting plasma glucose; HbA1c, glycosylated hemoglobin; HC, healthy control; NA, not applicable; NK cells, natural killer cells; T2DM, type 2 diabetes mellitus.

### 3.2 Antibody response after second dose of COVID‐19 vaccine

After the second dose of vaccine, the levels of anti‐SARS‐CoV‐2 total antibodies (1.57 vs. 3.43, *p* = 0.002; Figure S1A), anti‐RBD‐specific IgG (29.16 vs. 121.56, *p* = 0.0025; Figure S1B), and NAb toward WT (19.01 vs. 37.77, *p* = 0.0056; Figure S1C) were significantly lower than in patients with T2DM who had completed the booster vaccination. However, the NAb toward BA.4/5 level was not different from that of patients who had received three doses of the COVID‐19 vaccine (8.08 vs. 10.03, *p* = 0.12; Figure S1D).

### 3.3 Antibody response after third dose of COVID‐19 vaccine

All participants who had received the booster vaccination were divided into three groups according to the time interval between vaccination and sampling. At 0–3, 4–6, and >6 months, seropositivity rates of four detected antibodies were similar between patients with T2DM and HCs (Table 2).

**TABLE 2:** Seropositivity rates of SARS‐CoV‐2 antibodies after booster vaccination.

Antibody	Months after booster vaccination	HCs (*n* = 102)	T2DM (*n* = 184)	*p* value
Anti‐SARS‐CoV‐2 total antibodies^a^	0–3	100.0% (8/8)	100.0% (14/14)	1
	4–6	91.3% (21/23)	92.1% (35/38)	0.912
	>6	98.6% (70/71)	93.9% (124/132)	0.125
Anti‐RBD‐specific IgG^b^	0–3	100.0% (8/8)	100.0% (14/14)	1
	4–6	95.7% (22/23)	84.2% (32/38)	0.238
	>6	88.7% (63/71)	83.3% (110/132)	0.301
Neutralizing antibody (WT)^c^	0–3	100.0% (8/8)	100.0% (14/14)	1
	4–6	73.9% (17/23)	71.1% (27/38)	0.809
	>6	66.2% (47/71)	53.0% (70/132)	0.07
Neutralizing antibody (B.A.4/5)^c^	0–3	14.3% (1/7)	21.4% (3/14)	0.694
	4–6	4.3% (1/23)	5.3% (2/38)	0.873
	>6	7.0% (5/71)	9.1% (11/121)	0.62

Abbreviations: HC, healthy control; RBD, receptor‐binding domain; T2DM, type 2 diabetes mellitus; WT, wild type.

^a^ For anti‐SARS‐CoV‐2 total antibodies, optical density values above 0.19 were regarded as positive.

^b^ For anti‐RBD‐specific IgG, 11.6 BAU/ml was a threshold to divide sero‐positive and ‐negative samples.

^c^ The inhibition rate ≥ 30% was regarded as positive in neutralizing antibodies.

**FIGURE 1:**
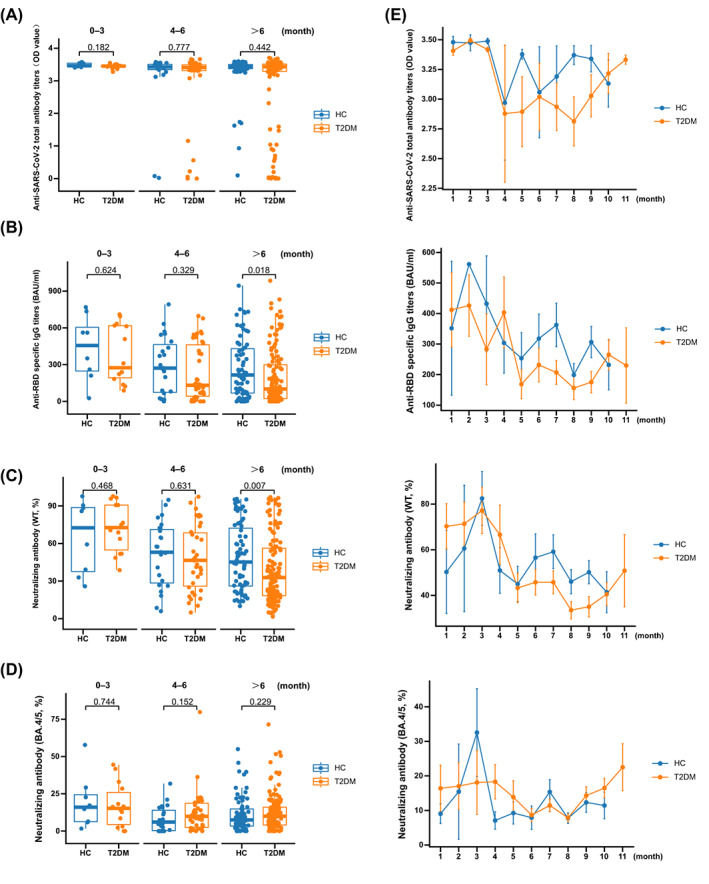
Antibody responses after booster COVID‐19 vaccination in HCs and patients with T2DM. The levels of anti‐SARS‐CoV‐2 total antibodies (A), anti‐RBD specific immunoglobulin G (IgG) (B), NAb toward WT (C), and NAb toward BA.4/5 (D) in HCs and patients with T2DM. The changes of anti‐SARS‐CoV‐2 total antibodies, anti‐RBD specific IgG, NAb toward WT, and NAb toward BA.4/5 levels over time in HCs and patients with T2DM after booster vaccination (E). COVID‐19, coronavirus disease 2019; HCs, healthy controls; T2DM, type 2 diabetes mellitus; SARS‐CoV‐2, severe acute respiratory syndrome coronavirus 2; RBD, receptor‐binding domain; NAb, neutralizing antibody; WT, wild type.

No significant differences were observed in total anti‐SARS‐CoV‐2 antibodies between patients with T2DM and HCs at 0–3, 4–6, and >6 months (Figure 1A; data are shown in Table S4). Meanwhile, the levels of anti‐SARS‐CoV‐2 total antibodies did not change in patients with T2DM even when the time after vaccination was prolonged (*p* = 0.281; Figure 1E).

The levels of anti‐RBD‐specific IgG did not significantly change between patients with T2DM and HCs at 0–3 and 4–6 months (Figure 1B; data are shown in Table S4), while a significantly decreased level was observed in patients with T2DM compared with HCs at >6 months (Figure 1B; data are shown in Table S4). The levels of anti‐RBD‐specific IgG were decreased with prolonged time after vaccination in patients with T2DM (*p* = 0.049; Figure 1E).

The inhibition rates of NAb toward WT were not significantly different between patients with T2DM and HCs at 0–3 and 4–6 months (Figure 1C; data are shown in Table S4), while a significantly downregulated inhibition rate was observed in patients with T2DM compared with HCs at >6 months (Figure 1C; data are shown in Table S4). The inhibition rates of NAb toward WT were decreased with prolonged time after vaccination in patients with T2DM (*p* = 0.002; Figure 1E).

For NAb toward BA.4/5, the inhibition rates showed no significant difference between patients with T2DM and HCs at 0–3, 4–6, and >6 months (Figure 1D; data are shown in Table S4). Meanwhile, the inhibition rates of NAb toward BA.4/5 did not change with prolonged time after vaccination in patients with T2DM (*p* = 0.133; Figure 1E).

### 3.4 Correlation of vaccination period and magnitude of SARS‐CoV‐2 antibodies after third dose

**FIGURE 2:**
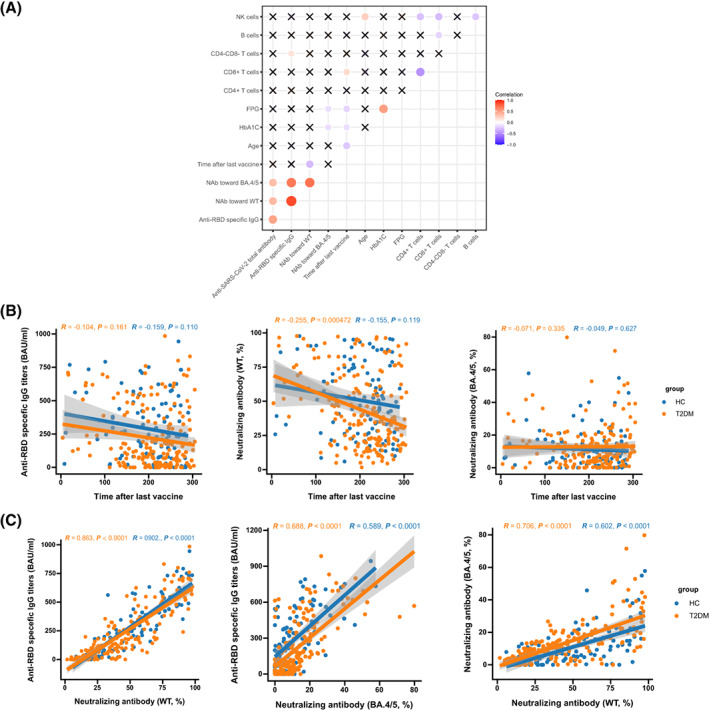
Correlation of vaccination period and the magnitude of SARS‐CoV‐2 antibodies after the third dose. (A) Correlation heatmap visualized the association between SARS‐CoV‐2 antibodies and clinical characteristics among T2DM. (B) Associations of immunoglobulin G (IgG anti‐RBD antibodies titers (BAU/ml), NAb toward WT (%), NAb toward BA.4/5 (%), and time after last vaccine. (C) Associations of IgG anti‐RBD antibodies (BAU/ml), NAb against WT (%) and NAb against BA.4/5 (%). COVID‐19, coronavirus disease 2019; HCs, healthy controls; T2DM, type 2 diabetes mellitus; SARS‐CoV‐2, severe acute respiratory syndrome coronavirus 2; RBD, receptor‐binding domain; NAb, neutralizing antibody; WT, wild type.

To further explore the association of antibody responses with clinical characteristics and laboratory parameters, we performed Spearman's correlation analyses. The levels of anti‐RBD‐specific IgG were positively associated with the proportions of CD3^+^CD4^−^CD8^−^ T cells (*r* = 0.150, *p* = 0.045; Figure 2A). The inhibition rates of NAb toward BA.4/5 were negatively related to the levels of FPG (*r* = −0.155, *p* = 0.035; Figure 2A) and HbA1c (*r* = −0.209, *p* = 0.004; Figure 2A). The inhibition rates of NAb toward WT were negatively correlated with time after the third dose in patients with T2DM (*r* = −0.255, *p* = 0.000472; Figure 2B). Furthermore, a robust and significant association was observed between anti‐RBD‐specific IgG and NAb toward WT (HCs: *r* = 0.902, *p* < 0.0001; T2DM: *r* = 0.863, *p* < 0.0001), NAb toward WT, and NAb toward BA.4/5 (HCs: *r* = 0.602, *p* < 0.0001; T2DM: *r* = 0.706, *p* < 0.0001), NAb toward BA.4/5 and anti‐RBD‐specific IgG in both HCs and patients with T2DM (HCs: *r* = 0.589, *p* < 0.0001; T2DM: *r* = 0.688, *p* < 0.0001) (Figure 2C).

### 3.5 Effect of clinical characteristics and laboratory parameters on antibody responses

To investigate the potential factors that affected the humoral responses to SARS‐CoV‐2 in patients with T2DM after receiving the third dose of the COVID‐19 vaccine, the effect of clinical characteristics and laboratory parameters on the seropositivity and levels of anti‐RBD‐specific IgG, NAb toward WT, and NAb toward BA.4/5 were analyzed. Analysis of clinical features showed no significant correlations between the seropositivity and variables such as age, gender, BMI, comorbidities, and diabetes complications (Tables 3 and S5). However, the seropositivity of anti‐RBD‐specific IgG was significantly correlated to the proportion of CD3^+^CD4^−^CD8^−^ T cells (*p* = 0.005; Table 3). In addition, the level of HbA1c was also significantly associated with the seropositivity of NAb toward BA.4/5 (*p* = 0.04; Table 3).

**TABLE 3:** Distribution of clinical characteristics by serum antibody titers to COVID‐19 vaccine in patients with T2DM.

Variables	Anti‐RBD‐IgG^a^		*p* value	Neutralizing antibody (WT)^b^		*p* value	Neutralizing antibody (BA.4/5)^b^		*p* value
	Negative (*n* = 28)	Positive (*n* = 156)		Negative (*n* = 73)	Positive (*n* = 111)		Negative (*n* = 168)	Positive (*n* = 16)	
Age (years)	66 (62–71)	66 (61–70)	0.684	65 (62–69)	66 (61–70)	0.924	66 (61–70)	63 (49–70)	0.233
<60, *n* (%)	4 (14.3%)	24 (15.4%)	0.882	11 (15.1%)	17 (15.3%)	0.964	23 (13.7%)	5 (31.2%)	0.062
≥60, *n* (%)	24 (85.7%)	132 (84.6%)		62 (84.9%)	94 (84.7%)		145 (86.3%)	11 (69.8%)	
Gender (male, *n* [%])	16 (57.1%)	86 (55.1%)	0.843	40 (54.8%)	62 (55.9%)	0.887	91 (54.2%)	11 (68.8%)	0.262
BMI (kg/m^2^)	25.43 (22.15–28.80)	24.89 (22.86–26.73)	0.515	25.25 (22.85–27.68)	24.89 (22.44–26.66)	0.421	24.99 (22.82–26.76)	24.56 (22.17–26.45)	0.618
<24, *n* (%)	9 (32.1%)	43 (27.6%)	0.348	22 (30.1%)	30 (27.0%)	0.599	47 (30.1%)	5 (31.3%)	0.824
24–28, *n* (%)	10 (25.7%)	76 (48.7%)		33 (45.2%)	53 (47.7%)		79 (50.6%)	7 (43.8%)	
≥28, *n* (%)	8 (28.6%)	25 (16.0%)		16 (21.9%)	17 (15.3%)		30 (19.2%)	4 (25.0%)	
FPG (mmol/L)	7.6 (6.7–9.3)	7.6 (6.8–9.7)	1	7.5 (6.8–8.7)	7.6 (6.9–8.8)	0.843	7.6 (6.8–8.8)	7.7 (7.1–9.2)	0.753
HbA1c (%)	7.0 (6.5–7.8)	7.1 (6.6–7.8)	0.995	6.9 (6.5–7.9)	7.1 (6.7–7.7)	0.642	7.1 (6.6–7.8)	6.7 (6.5–7.1)	0.04
CD3^+^CD4^+^ T cells (% of lymphocytes)	39.32 (34.53–44.93)	37.71 (32.72–44.78)	0.545	38.54 ± 9.03	38.87 ± 7.50	0.793	38.01 (32.74–45.00)	37.03 (32.67–44.50)	0.725
CD3^+^CD8^+^ T cells (% of lymphocytes)	22.25 (18.50–32.90)	23.72 (17.90–29.01)	0.858	24.49 (18.47–32.84)	23.16 (17.84–28.20)	0.357	23.51 (17.95–29.35)	23.43 (18.62–32.43)	0.769
CD3^+^CD4^−^CD8^−^ T cells (% of lymphocytes)	2.62 (1.70–3.68)	3.54 (2.33–5.14)	0.005	3.14 (2.05–4.74)	3.53 (2.35–5.00)	0.331	3.30 (2.12–4.76)	4.15 (3.52–5.22)	0.068
NK cells (% of lymphocytes)	17.75 (11.34–25.02)	16.83 (11.73–24.10)	0.816	17.59 (11.95–23.91)	16.71 (11.71–24.27)	0.834	17.24 (12.41–24.10)	12.62 (8.85–25.90)	0.375
B cells (% of lymphocytes)	10.18 (6.77–15.22)	10.98 (8.09–13.69)	0.47	10.43 (7.33–13.70)	11.17 (8.36–13.87)	0.299	10.94 (7.76–13.74)	10.47 (7.22–13.76)	0.762
Comorbidities									
Hypertension	18 (64.3%)	96 (61.5%)	0.783	42 (57.5%)	72 (64.9%)	0.153	104 (61.9%)	10 (62.5%)	0.963
Hyperlipemia	20 (71.4%)	117 (75.0%)	0.69	55 (75.3%)	83 (74.8%)	0.931	124 (73.8%)	14 (87.5%)	0.365
Chronic respiratory disease	2 (7.2%)	19 (12.2%)	0.44	9 (12.3%)	12 (10.8%)	0.751	18 (10.7%)	3 (18.8%)	0.401
Cardiovascular and cerebrovascular diseases	14 (50.0)	84 (53.8%)	0.707	40 (54.8%)	59 (53.2%)	0.827	88 (52.4%)	11 (68.8%)	0.295
Liver diseases	5 (17.9%)	15 (9.6%)	0.197	9 (12.3%)	11 (9.9%)	0.606	16 (9.5%)	4 (25.0%)	0.078
Kidney diseases	4 (14.3%)	14 (9.0%)	0.384	8 (11.0%)	10 (9.0%)	0.663	18 (10.7%)	0	0.373
Autoimmune diseases	0	2 (1.3%)	0.547	1 (1.4%)	1 (0.9%)	1.000	2 (1.2%)	0	1
Cancer	1 (3.6%)	5 (3.2%)	0.92	2 (2.7%)	4 (3.6%)	1.000	6 (3.6%)	0	1

Abbreviations: BMI, body mass index; FPG, fasting plasma glucose; HbA1c, glycosylated hemoglobin; NK cells, natural killer cells; RBD, receptor‐binding domain; T2DM, type 2 diabetes mellitus; WT, wild type.

^a^ For anti‐RBD‐specific IgG, 11.6 BAU/ml was a threshold to divide sero‐positive and ‐negative samples.

^b^ The inhibition rate ≥ 30% was regarded as positive in neutralizing antibodies.

**FIGURE 3:**
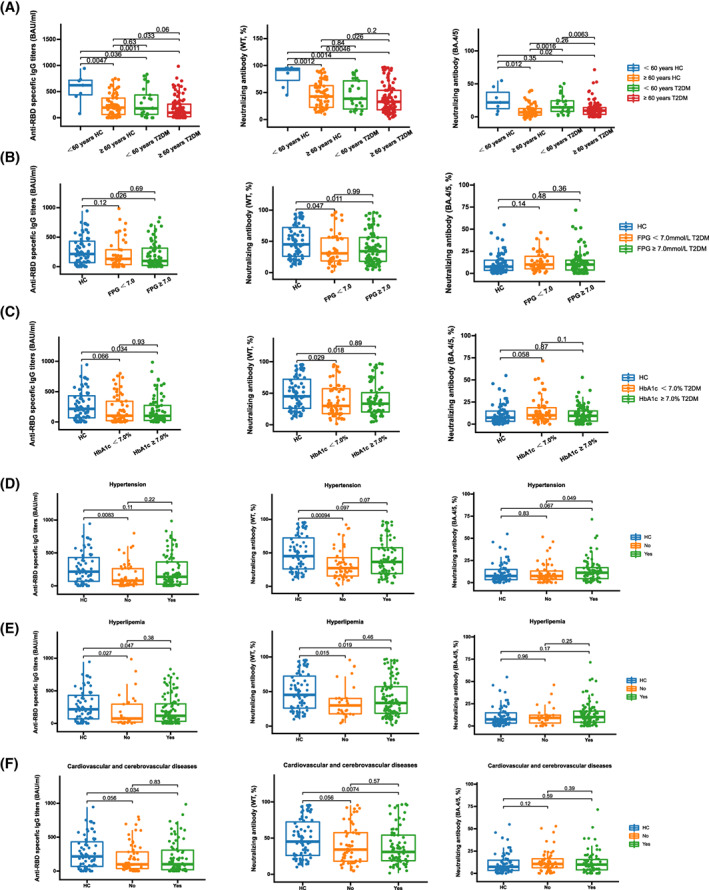
Association analysis between clinical characteristics and poor antibody responses to COVID‐19 vaccine after booster vaccination more than 6 months in patients with T2DM. (A) Age in patients with T2DM. (B) FPG in patients with T2DM. (C) HbA1c in patients with T2DM, (D) Hypertension in patients with T2DM. (E) Hyperlipemia in patients with T2DM. (F) Cardiovascular and cerebrovascular diseases in patients with T2DM. COVID‐19, coronavirus disease 2019; HCs, healthy controls; T2DM, type 2 diabetes mellitus; FPG, fasting plasma glucose; HbA1c, hemoglobin A1c.

Furthermore, the associations between the levels of three antibodies after a booster vaccination that was received more than 6 months and clinical characteristics and laboratory parameters were analyzed. The inhibition rates of NAb toward BA.4/5 were significantly lower in patients with T2DM aged ≥60 years compared to younger patients (*p* = 0.0063; Figure 3A). No significant differences were found for any SARS‐CoV‐2 antibodies between patients with T2DM whose FPG was <7.0 mmol/L and those whose FPG was ≥7.0 mmol/L, while the levels of anti‐RBD‐specific IgG (*p* = 0.026; Figure 3B) and inhibition rates of NAb toward WT (*p* = 0.011; Figure 3B) were significantly lower in patients with T2DM whose FPG was ≥7.0 mmol/L compared with HCs. Similar results were observed in patients with T2DM whose HbA1c was ≥7.0% (anti‐RBD‐specific IgG: *p* = 0.034 [Figure 3C]; NAb toward WT: *p* = 0.018 [Figure 3C]). The inhibition rates of NAb toward BA.4/5 were significantly increased in patients with T2DM who had hypertension compared with those without (*p* = 0.049; Figure 3D). The levels of anti‐RBD‐specific IgG (T2DM with hyperlipemia: *p* = 0.047 [Figure 3E]; T2DM without hyperlipemia: *p* = 0.027 [Figure 3E]) and inhibition rates of NAb toward WT (T2DM with hyperlipemia: *p* = 0.019 [Figure 3E]; T2DM without hyperlipemia: *p* = 0.015 [Figure 3E]) were significantly decreased in both patients with T2DM who had hyperlipemia and those without compared with HCs. Similar results were found in patients with T2DM who had cardiovascular and cerebrovascular diseases (anti‐RBD‐specific IgG: *p* = 0.034 [Figure 3F]; NAb toward WT: *p* = 0.0074 [Figure 3F]).

## 4 DISCUSSION

In the present study, we investigated the humoral response of HCs and patients with T2DM after a booster shot of inactivated COVID‐19 vaccine by detecting anti‐SARS‐CoV‐2 total antibodies, anti‐RBD‐specific IgG, NAb toward WT, and BA.4/5. The data showed that patients with T2DM had a reduced antibody response after receiving the third dose of the COVID‐19 vaccine for more than 6 months (Table S4), while no significant difference of antibody response was found between HCs and patients with T2DM after boost immunization within 6 months (Table S4). Furthermore, both HCs and patients with T2DM were unable to avoid infection with BA.4/5 subvariants because their antibody titers of NAb toward BA.4/5 were still below the minimal inhibition rate of NAb toward BA.4/5, regardless of the time after booster vaccination (Table S4).

To our knowledge, our study is the first to investigate the antibody response in patients with T2DM after receiving the third dose of inactivated COVID‐19 vaccines. Several studies have observed the impaired antibody response of the second dose of inactivated COVID‐19 vaccines. Xiang et al. reported that the levels of anti‐RBD‐IgG and NAbs were significantly lower in patients with T2DM compared to HCs after full‐course inactivated COVID‐19 vaccines.^12^ Meanwhile, Cheng et al. revealed decreased titers of anti‐N/S IgG and anti‐RBD IgG in patients with T2DM compared to HCs.^22^ In addition, numerous studies also indicated reduced immunogenicity to mRNA or viral vector‐based COVID‐19 vaccines after finishing full‐course immunization.^4^, ^5^, ^13^ Therefore, it is necessary for patients with T2DM to acquire a booster vaccination to improve the effectiveness of the vaccine.^14^ Sourij et al demonstrated a higher antibody response in patients with T2DM inoculated with the third dose of COVID‐19 mRNA vaccine than with the second dose, and response to the third vaccination showed no significant difference between patients with T2DM and HCs.^5^ In this study, we also observed enhanced humoral reactivity in patients with T2DM after the booster dose of inactivated vaccine compared to those after receiving the second dose of vaccine, including anti‐SARS‐CoV‐2 total antibodies (*p* = 0.002; Figure S1A), anti‐RBD IgG (*p* = 0.0025; Figure S1B), and NAb toward WT (*p* = 0.0056; Figure S1C). Furthermore, a similar antibody response to inactivated COVID‐19 vaccine was shown in HCs and patients with T2DM within 6 months of booster vaccination, indicating that booster vaccination helps enhance the protection by immunization.

Anti‐SARS‐CoV‐2 total antibodies show no differences between HCs and patients with T2DM after receiving the third dose of inactivated COVID‐19 vaccine (Figure 1A). Furthermore, no correlation was found between the levels of anti‐SARS‐CoV‐2 total antibodies and time after the third vaccine dose (HCs: *r* = −0.58, *p* = 0.878; T2DM: *r* = −0.091, *p* = 0.218). However, the inhibition rate of NAb toward WT was significantly lower in patients with T2DM after they had received the third dose of inactivated COVID‐19 vaccine more than 6 months later, and a significant association was observed between the inhibition rate of NAb toward WT and time after the third dose of vaccine in patients with T2DM (*r* = −0.255, *p* = 0.000472). Therefore, the majority of anti‐SARS‐CoV‐2 total antibodies might be not NAbs, and the protective immunity of the inactivated COVID‐19 vaccine is impaired after a booster shot vaccine for more than 6 months. Anti‐RBD IgG is regarded as neutralizing against SARS‐CoV‐2,^15^, ^16^ which was positively correlated with the inhibition rates of NAb toward WT in this study (HC: *r* = 0.902, *p* < 0.0001; T2DM: *r* = 0.863, *p* < 0.0001). The levels of anti‐RBD IgG were also decreased in patients with T2DM compared to HCs after the third dose of vaccine for more than 6 months, which is consistent with the difference of NAb toward WT in patients with T2DM. To summarize, the antibodies to neutralize SARS‐CoV‐2 were impaired in patients with T2DM after receiving the third dose of vaccine for more than 6 months; therefore, an additional vaccine dose could be administered due to waning immunoreactivity at 6–8 months following vaccination.^17^, ^18^

Omicron variants have become dominant epidemic strains in many countries. To date, global surveillance data suggest higher transmissibility of BA.4/5 than previous VOCs and WT strains.^9^ The pathogenicity of Omicron variants is attenuated compared to Delta and the original strain.^19^ However, patients with diabetes have a higher risk for deterioration of SARS‐CoV‐2 Omicron variant infection.^20^ Our study showed that both HCs and patients with T2DM were unable to defend themselves against BA.4/5 infection due to a lower‐than‐threshold inhibition rate of NAb toward BA.4/5, indicating that breakthrough infection of Omicron variants might occur although these individuals had completed booster immunization. Nevertheless, booster vaccination still plays an important role in protecting against symptomatic infection and severe disease from Omicron subvariants.^21^ Therefore, there exists an urgent necessity for a booster vaccination to enhance protection against Omicron subvariants.

Some risk factors affect the antibody response to the COVID‐19 vaccine. Glycemic control is essential to avoid severe complications for patients with T2DM. However, the influence of FPG in serological response toward the COVID‐19 vaccine remains unclear in patients with T2DM. For patients with T2DM who received the second inactivated dose, Cheng et al. found that those with FPG ≥ 7 mmol/L achieved lower seroconversion rates than those with well‐controlled FPG.^22^ Another study showed that there was no significant difference in anti‐RBD IgG titers between patients with T2DM with FPG ≥ 7 mmol/L and those without.^12^ Our results consistently revealed no significant change in anti‐RBD‐specific IgG levels between patients with T2DM with FPG ≥ 7.0 mmol/L and those with well‐controlled FPG. Meanwhile, we did not observe a significant difference in anti‐RBD‐specific IgG levels and the inhibition rates of NAb toward WT and BA.4/5 between patients with T2DM whose HbA1c was ≥7.0% and those without, while those with HbA1c ≥ 7.0% achieved lower seropositivity of NAb toward BA.4/5 than patients with T2DM with well‐controlled HbA1c. Therefore, glycemic control is still beneficial for patients with T2DM to prevent poor prognosis when they are infected with SARS‐CoV‐2. Additionally, we found the seropositivity of anti‐RBD‐specific IgG was significantly correlated to the proportion of CD3^+^CD4^−^CD8^−^ T cells. CD3 + CD4^−^CD8^−^ T cells are considered regulatory T lymphocytes,^23^ which have been found significantly decreased in nonsurvivors of COVID‐19 and progressively reduced from mild to severe COVID‐19 patients.^24^ The roles of CD3^+^CD4^−^CD8^−^ T cells in antibody response to the COVID‐19 vaccine need to be further examined.

It should be noted that most of the participants in this study were elderly people who were over 60 years old. Elderly individuals with decreased immunity are susceptible to COVID‐19 infection and more prone to severe disease and death compared to young people,^25^, ^26^, ^27^ indicating that they should strengthen protection to avoid infection.^28^ Furthermore, the elderly showed poorer vaccine efficiency compared to younger people who received the COVID‐19 vaccine due to impaired antibody response and T cell response.^29^, ^30^, ^31^ Those who had lower antibody response after receiving the COVID‐19 vaccine were recommended to further receive the vaccine to enhance immunity.^32^ Meanwhile, the majority of patients with diabetes in China are elderly. Therefore, it is necessary to understand the effect of the COVID‐19 vaccine on the elderly population with diabetes, as the effect of the vaccine is poor in them and they are susceptible to SARS‐CoV‐2, in order to identify those individuals who really need to be further vaccinated and to reduce the risk of infection and development of severe COVID‐19 in immunocompromised or aged people. In our study, we observed that the inhibition rates of NAb toward BA.4/5 were significantly lower in patients with T2DM aged ≥60 years compared to young patients (*p* = 0.0063; Figure 3A), indicating that elderly patients were more susceptibility to infection with the Omicron variant, and further vaccination should be considered in those with low immunity.

There are two main limitations of this study. On the one hand, this study was of a cross‐sectional design, so we could not monitor the dynamic changes of antibody levels for each time point after booster vaccination because serial samples of participants were not accessible. On the other hand, we only investigated the humoral response to inactivated COVID‐19 vaccine, and we could not detect cellular immunity toward the COVID‐19 vaccine due to the limited blood sample volume we collected. Cellular immune data contribute to a complete understanding of the effect of inactivated COVID‐19 vaccine. Therefore, further research on cellular responses in patients with T2DM after the booster dose of inactivated vaccine is needed.

## 5 CONCLUSION

In conclusion, after being vaccinated with the third dose of inactivated COVID‐19 vaccines for more than 6 months, the levels of anti‐RBD‐specific IgG and inhibition rate of NAb toward WT significantly decreased in patients with T2DM compared to HCs. The fact that both HCs and T2DM showed a decreased inhibition rate of NAb toward BA.4/5 compared to WT gives rise to the possibility of breakthrough infections. Considering the reduction of antibody response after booster vaccination for more than 6 months in patients with T2DM, a further booster shot may be needed to maintain protective immunity.

## AUTHOR CONTRIBUTIONS

All authors significantly contributed to the manuscript and approved of the final version for publication. Yongzhe Li and Xinhua Xiao conceived and designed the study. Haolong Li, Yao Wang, Xiaomeng Li, Xinxin Feng, and Siyu Wang contributed to sample collection and data acquisition. Haolong Li and Xiaomeng Li carried out the statistical analysis. All authors contributed to interpretation of data. Haolong Li wrote the first manuscript draft, which was critically revised by Yao Wang, Yongzhe Li, and Xinhua Xiao.

## FUNDING INFORMATION

This work was supported by the Beijing Natural Science Foundation (M23008), National Key Research and Development Program of China (2018YFE0207300), Beijing Municipal Science & Technology Commission (Z211100002521021), and the National High Level Hospital Clinical Research Funding (2022‐PUMCH‐B‐124).

## DISCLOSURE

The authors declare that they have no conflict of interest.

## Supporting information


**FIGURE S1.** Comparison of antibody response between full‐course and booster COVID‐19 vaccination in HCs and patients with T2DM. The levels of anti‐SARS‐CoV‐2 total antibodies (A), anti‐RBD‐specific IgG (B), NAb toward WT (C), and NAb toward BA.4/5 (D) in HCs and patients with T2DM. COVID‐19, coronavirus disease 2019; HCs, healthy controls; T2DM, type 2 diabetes mellitus; SARS‐CoV‐2, severe acute respiratory syndrome coronavirus 2; RBD, receptor‐binding domain; NAb, neutralizing antibody; WT, wild type.

**TABLE S1.** Medications in HCs and patients with T2DM. HCs, healthy controls; T2DM, type 2 diabetes mellitus.

**TABLE S2.** The characteristics of participants are based on different duration after the third dose of the COVID‐19 vaccine.

**TABLE S3.** The clinical characteristic of patients with type 2 diabetes mellitus (T2DM) between different vaccination status.

**TABLE S4.** Comparison of SARS‐CoV‐2 antibody titers after booster vaccination.

**TABLE S5.** Comparison of SARS‐CoV‐2 antibody titers between type 2 diabetes mellitus (T2DM) subgroups.

## Data Availability

The data are available from the corresponding author upon reasonable request.
