# Supplementary material for: Antibody response to inactivated COVID‐19 vaccine in patients with type 2 diabetes mellitus after the booster immunization
Source: J Diabetes. 2023 Jul 30;15(11):931–43. doi: 10.1111/1753-0407.13448 (PMC10667667; doi:10.1111/1753-0407.13448)
Supplement: Supplementary file 1 — FIGURE S1. Comparison of antibody response between full‐course and booster COVID‐19 vaccination in HCs and patients with T2DM. The levels of anti‐SARS‐CoV‐2 total antibodies (A), anti‐RBD‐specific IgG (B), NAb toward WT (C), and NAb toward BA.4/5 (D) in HCs and patients with T2DM. COVID‐19, coronavirus disease 2019; HCs, healthy controls; T2DM, type 2 diabetes mellitus; SARS‐CoV‐2, severe acute respiratory syndrome coronavirus 2; RBD, receptor‐binding domain; NAb, neutralizing antibody; WT, wild type. [file JDB-15-931-s002.pdf]

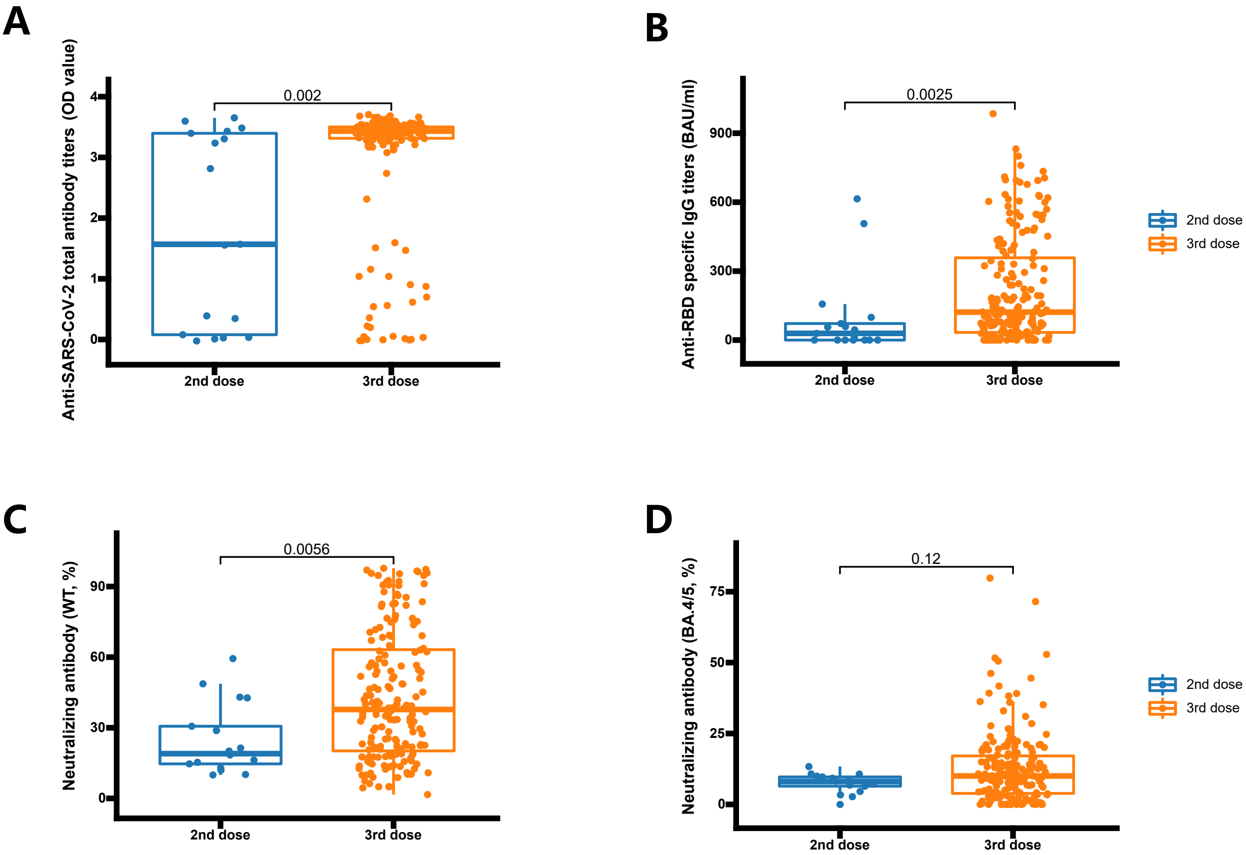

Figure S1. Comparison of antibody response between full-course and booster COVID-19 vaccination in HCs and patients with T2DM. The levels of anti-SARS-CoV-2 total antibody (A), anti-RBD specific IgG (B), NAb toward WT (C), and NAb toward BA.4/5 (D) in HCs and patients with T2DM. HCs, healthy controls; T2DM, type 2 diabetes mellitus; NAb, neutralizing antibody; WT, wild type.
