# Supplementary material for: Antibody response to inactivated COVID‐19 vaccine in patients with type 2 diabetes mellitus after the booster immunization
Source: J Diabetes. 2023 Jul 30;15(11):931–43. doi: 10.1111/1753-0407.13448 (PMC10667667; doi:10.1111/1753-0407.13448)
Supplement: Supplementary file 2 — TABLE S1. Medications in HCs and patients with T2DM. HCs, healthy controls; T2DM, type 2 diabetes mellitus. [file JDB-15-931-s005.docx]

Table S1. Medications in HCs and patients with T2DM.

|  | HCs (n = 102) | T2DM (n = 201) |
| --- | --- | --- |
| Treatment received, N (%) |  |  |
| α-Glucosidase inhibitors | NA | 41 (20.4%) |
| Metformin | NA | 124 (61.7%) |
| Sulfonylureas | NA | 45 (22.4%) |
| Insulins | NA | 55(27.4%) |
| Dipeptidyl peptidase-4 inhibitors | NA | 61 (30.3%) |
| Glucagon-like peptide-1 receptor agonist | NA | 11 (5.5%) |
| Sodium-dependent glucose transporters 2 | NA | 53 (26.4%) |
| Thiazolidinedione | NA | 4 (2.0%) |
| Statin | NA | 49 (24.4%) |
| Ezetimibe | NA | 6 (3.0%) |
| Aspirin | NA | 24 (11.9%) |
| Adenosine diphosphate receptor antagonist | NA | 1(0.4%) |
| Angiotensin II receptor antagonists | NA | 18 (9.0%) |
| Calcium channel blockers | NA | 20 (10.0%) |
| Compound methoxyphenamine | NA | 1 (0.5%) |
| Coumarin anticoagulants | NA | 1 (0.5%) |
| Isosorbide-5-mononitrate | NA | 1 (0.5%) |
| loop diuretics | NA | 2 (1.0%) |
| Mecobalamin | NA | 15 (7.5%) |
| Non-steroidal anti-inflammatory drug | NA | 1 (0.5%) |
| Spironolactone | NA | 2 (1.0%) |
| β1-receptor blocker | NA | 14 (7.0%) |
| Untreated | 102 (100.0%) | 20 (10.0%) |

Abbreviations: HCs, healthy controls; T2DM, type 2, type 2 diabetes mellitus.
