# Supplementary material for: Antibody response to inactivated COVID‐19 vaccine in patients with type 2 diabetes mellitus after the booster immunization
Source: J Diabetes. 2023 Jul 30;15(11):931–43. doi: 10.1111/1753-0407.13448 (PMC10667667; doi:10.1111/1753-0407.13448)
Supplement: Supplementary file 3 — TABLE S2. The characteristics of participants are based on different duration after the third dose of the COVID‐19 vaccine. [file JDB-15-931-s004.docx]

Table S2. The characteristics of participants are based on different duration after the third dose of the COVID-19 vaccine.

| Variables | 0-3 month |  |  | 4-6 month |  |  | ＞ 6 month |  | P value |
| --- | --- | --- | --- | --- | --- | --- | --- | --- | --- |
|  | HCs (n = 8) | T2DM (n = 14) | P value | HCs (n = 23) | T2DM (n = 38) | P value | HCs (n = 71) | T2DM (n = 132) | 0.17 |
| Age (years) | 65 (62-70) | 68 (63-72) | 0.793 | 64 (62-67) | 66 (63-68) | 0.279 | 64 (61-67) | 65 (61-70) | 0.09 |
| ＜ 60, n (%) | 0 | 1 (7.1%) | 1 | 3 (13.0%) | 2 (5.3%) | 0.356 | 7 (9.8%) | 25 (18.9%) |  |
| ≥ 60, n (%) | 8 (100.0%) | 13 (92.9%) |  | 20 (87.0%) | 36 (94.7%) |  | 64 (9.0%) | 107 (81.1%) | 0.971 |
| Gender (male, n (%)) | 4 (50.0%) | 11 (78.6%) | 0.343 | 14 (60.9%) | 20 (52.6%) | 0.53 | 38 (53.5%) | 71 (53.8%) | 0.068 |
| BMI (kg/m2) | 25.72 (23.25-28.78) | 24.65 (22.19-25.58) | 0.238 | 24.61 (21.59-26.90) | 24.39 (22.64-25.96) | 0.864 | 24.16 (22.55-26.25) | 25.31 (22.84-27.44) | 0.014 |
| <24, n (%) | 3 (37.5%) | 3 (21.4%) | 0.112 | 7 (30.4%) | 12 (31.6%) | 0.787 | 34 (47.9%) | 37 (28.0%) |  |
| 24–28, n (%) | 3 (37.5%) | 9 (64.3%) |  | 7 (30.4%) | 17 (44.7%) |  | 28 (39.4%) | 60 (45.5%) |  |
| ≥28, n (%) | 2 (25.0%) | 0 |  | 4 (17.4%) | 6 (15.8%) |  | 7 (9.8%) | 27 (20.5%) | ＜0.001 |
| FPG (mmol/L) | 5.58±0.47 | 8.41±1.63 | ＜0.001 | 5.35±0.34 | 8.42±2.23 | ＜0.001 | 5.4 (5.1-5.7) | 7.4 (6.7-8.6) | ＜0.001 |
| HbA1c (%) | 5.7 (5.5-6.0) | 7.1 (6.7-7.5) | ＜0.001 | 5.6 (5.4-5.8) | 7.1 (6.8-7.7) | ＜0.001 | 5.6 (5.4-5.8) | 7.0 (6.6-7.8) | 0.682 |
| CD3+CD4+ T cells (% of lymphocytes) | 40.65±9.19 | 36.72±7.22 | 0.278 | 26.25±9.63 | 41.44±8.89 | 0.054 | 36.86 (32.01-45.51) | 37.63 (32.67-44.10) | 0.975 |
| CD3+CD8+ T cells (% of lymphocytes) | 22.78±10.54 | 23.85±9.99 | 0.814 | 22.63 (20.30-29.75) | 21.76 (18.07-30.32) | 0.463 | 23.58 (18.12-30.84) | 23.88 (17.99-29.43) | 0.007 |
| CD3+CD4-CD8- T cells (% of lymphocytes) | 2.70±1.31 | 3.69±2.44 | 0.302 | 2.81 (2.02-5.19) | 3.04 (2.10-4.83) | 0.862 | 4.14 (2.96-6.23) | 3.43 (2.31-4.89) | 0.586 |
| NK cells (% of lymphocytes) | 21.62 (11.22-30.47) | 18.51 (11.43-29.58) | 0.977 | 19.38 (16.88-27.93) | 17.24 (11.81-21.75) | 0.095 | 16.36 (10.63-23.57) | 16.83 (11.80-24.30) | 0.170 |
| B cells (% of lymphocytes) | 9.79±3.07 | 11.51±4.54 | 0.352 | 9.75±3.45 | 10.95±5.11 | 0.373 | 9.64 (7.66-12.95) | 10.98 (7.85-14.16) |  |
| Comorbidities |  |  |  |  |  |  |  |  | NA |
| Hypertension | 0 | 8 (57.1%) | NA | 0 | 23 (60.5%) | NA | 0 | 82 (62.1%) | NA |
| Hyperlipemia | 0 | 10 (71.4%) | NA | 0 | 25 (65.8%) | NA | 0 | 102 (77.3%) | NA |
| Chronic respiratory disease | 0 | 0 | NA | 0 | 6 (15.8%) | NA | 0 | 15 (11.4%) | NA |
| Cardiovascular and cerebrovascular diseases | 0 | 8 (57.1%) | NA | 0 | 15 (39.5%) | NA | 0 | 74 (56.1%) | NA |
| Liver diseases | 0 | 1 (7.1%) | NA | 0 | 4 (10.5%) | NA | 0 | 15 (11.4%) | NA |
| Kidney diseases | 0 | 0 | NA | 0 | 6 (15.8%) | NA | 0 | 12 (9.1%) | NA |
| Autoimmune diseases | 0 | 0 | NA | 0 | 0 | NA | 0 | 2 (1.5%) | NA |
| Cancer | 0 | 0 | NA | 0 | 2 (5.3%) | NA | 0 | 4 (3.0%) |  |
| Period of the third vaccination at the time of sampling (day) | 45±32 | 45±23 | 0.982 | 142±25 | 145±22 | 0.557 | 237±31 | 240±34 | NA |

Abbreviations: HCs, healthy controls; T2DM, type 2 diabetes mellitus; BMI, body mass index; FPG, fasting plasma glucose; HbA1c, hemoglobin A1c; NK cells, natural killer cells.
