# Supplementary material for: Antibody response to inactivated COVID‐19 vaccine in patients with type 2 diabetes mellitus after the booster immunization
Source: J Diabetes. 2023 Jul 30;15(11):931–43. doi: 10.1111/1753-0407.13448 (PMC10667667; doi:10.1111/1753-0407.13448)
Supplement: Supplementary file 4 — TABLE S3. The clinical characteristic of patients with type 2 diabetes mellitus (T2DM) between different vaccination status. [file JDB-15-931-s006.docx]

Table S3. The clinical characteristic of patients with T2DM between different vaccination status.

| Variables | After 2nd dose (n = 17) | After 3rd dose (n = 184) | *P* value |
| --- | --- | --- | --- |
| Age (years) | 71 (60-81) | 66 (61-70) | 0.04 |
| ＜ 60, n (%) | 4 (23.5%) | 28 (15.2%) | 0.37 |
| ≥ 60, n (%) | 13 (76.5%) | 156 (84.8%) |  |
| Gender (male, n (%)) | 7 (41.2%) | 102 (55.4%) | 0.259 |
| BMI (kg/m2) | 23.76 (19.48-26.84) | 25.30 (23.53-26.95) | 0.097 |
| <24, n (%) | 7 (41.2%) | 52 (30.4%) | 0.254 |
| 24–28, n (%) | 6 (35.3%) | 86 (50.3%) |  |
| ≥28, n (%) | 1 (5.9%) | 33 (19.3%) |  |
| FPG (mmol/L) | 7.8 (6.9-10.7) | 7.60 (6.80-8.78) | 0.331 |
| HbA1c (%) | 8.8 (7.2-9.3) | 7.0 (6.6-7.8) | 0.003 |
| CD3+CD4+ T cells (% of lymphocytes) | 39.17 (28.72-48.24) | 37.71 (32.74-44.78) | 0.884 |
| CD3+CD8+ T cells (% of lymphocytes) | 22.48 (17.28-26.77) | 23.51 (17.95-29.35) | 0.315 |
| CD3+CD4-CD8- T cells (% of lymphocytes) | 3.04 (1.96-6.39) | 3.40 (2.27-4.86) | 0.855 |
| NK cells (% of lymphocytes) | 16.80 (11.96-23.59) | 17.22 (11.73-24.15) | 0.947 |
| B cells (% of lymphocytes) | 11.30 (8.27-15.60) | 10.94 (7.76-13.74) | 0.657 |
| Comorbidities |  |  |  |
| Hypertension | 8 (47.1%) | 114 (62.0%) | 0.229 |
| Hyperlipemia | 9 (52.9%) | 138 (75.0%) | 0.05 |
| Chronic respiratory disease | 2 (11.8%) | 21 (11.4%) | 1 |
| Cardiovascular and cerebrovascular diseases | 8 (47.1%) | 99 (53.8%) | 0.594 |
| Liver diseases | 2 (11.8%) | 20 (10.9%) | 1 |
| Kidney diseases | 2 (11.8%) | 18 (9.8%) | 0.68 |
| Autoimmune diseases | 0 | 2 (1.1%) | 1 |
| Cancer | 0 | 6 (3.3%) | 1 |

Abbreviations: BMI, body mass index; FPG, fasting plasma glucose; HbA1c, hemoglobin A1c; NK cells, natural killer cells.
