# Supplementary material for: Antibody response to inactivated COVID‐19 vaccine in patients with type 2 diabetes mellitus after the booster immunization
Source: J Diabetes. 2023 Jul 30;15(11):931–43. doi: 10.1111/1753-0407.13448 (PMC10667667; doi:10.1111/1753-0407.13448)
Supplement: Supplementary file 5 — TABLE S4. Comparison of SARS‐CoV‐2 antibody titers after booster vaccination. [file JDB-15-931-s003.docx]

Table S4. Comparison of SARS-CoV-2 antibody titers after booster vaccination.

| Antibody | Month after booster vaccination | HCs | T2DM | *P* value |
| --- | --- | --- | --- | --- |
| Anti-SARS-CoV 2 total antibody | 0 - 3 (n=8 vs n=14) | 3.48±0.06 | 3.44±0.07 | 0.182 |
|  | 4--6 (n=23 vs n=38) | 3.42 (3.34-3.53) | 3.41 (3.31-3.49) | 0.777 |
|  | ＞ 6 (n=71 vs n=132) | 3.43 (3.37-3.51) | 3.43 (3.29-3.51) | 0.442 |
| Anti-RBD specific IgG | 0 - 3 (n=8 vs n=14) | 434.30±264.06 | 380.13±235.07 | 0.624 |
|  | 4--6 (n=23 vs n=38) | 271.84 (66.72-488.63) | 131.17 (35.95-479.88) | 0.329 |
|  | ＞ 6 (n=71 vs n=132) | 215.46 (67.41-438.57) | 101.53 (21.40-305.91) | 0.018 |
| Neutralizing antibody (WT) | 0 - 3 (n=8 vs n=14) | 64.92±29.17 | 72.63±19.82 | 0.468 |
|  | 4--6 (n=23 vs n=38) | 51.32±26.13 | 48.00±25.95 | 0.631 |
|  | ＞ 6 (n=71 vs n=132) | 45.19 (25.70-73.98) | 32.76 (18.12-56.41) | 0.007 |
| Neutralizing antibody (B.A.4/5) | 0 - 3 (n=8 vs n=14) | 19.46±18.01 | 17.11±14.79 | 0.744 |
|  | 4--6 (n=23 vs n=38) | 6.02 (0-15.70) | 9.87 (2.08-19.27) | 0.152 |
|  | ＞ 6 (n=71 vs n=132) | 7.45 (3.19-14.88) | 9.92 (4.02-16.28) | 0.229 |
