# Supplementary material for: Antibody response to inactivated COVID‐19 vaccine in patients with type 2 diabetes mellitus after the booster immunization
Source: J Diabetes. 2023 Jul 30;15(11):931–43. doi: 10.1111/1753-0407.13448 (PMC10667667; doi:10.1111/1753-0407.13448)
Supplement: Supplementary file 6 — TABLE S5. Comparison of SARS‐CoV‐2 antibody titers between type 2 diabetes mellitus (T2DM) subgroups. [file JDB-15-931-s001.docx]

Table S5. Comparison of SARS-CoV-2 antibody titers between T2DM subgroups.

| Antibody | Characteristic | Positivity | P value |
| --- | --- | --- | --- |
| Anti-RBD specific IgG | HbA1C |  |  |
|  | ＜ 7.0% (n = 58) | 79.3% (46/58) | 0.288 |
|  | ≥ 7.0% (n = 73) | 86.3% (63/73) |  |
|  | FPG |  |  |
|  | ＜ 7.0 mmol/L (n = 43) | 81.4% (35/43) | 0.678 |
|  | ≥ 7.0 mmol/L (n = 89) | 84.3% (75/89) |  |
|  | Hypertension |  |  |
|  | Yes (n = 82) | 82.9% (68/82) | 0.876 |
|  | No (n = 50) | 84.0% (42/50) |  |
|  | Hyperlipemia |  |  |
|  | Yes (n = 102) | 83.3% (85/102) | 1 |
|  | No (n = 30) | 83.3% (25/30) |  |
|  | Cardiovascular and cerebrovascular diseases |  |  |
|  | Yes (n = 74) | 81.1% (60/74) | 0.433 |
|  | No (n = 58) | 86.2% (50/58) |  |
|  | Insulin treatment |  |  |
|  | Yes (n = 32) | 87.5% (28/32) | 0.467 |
|  | No (n = 100) | 82.0% (82/100) |  |
| Neutralizing antibody (WT) | HbA1C |  |  |
|  | ＜ 7.0% (n = 58) | 50.0% (29/58) | 0.482 |
|  | ≥ 7.0% (n = 73) | 56.2% (41/73) |  |
|  | FPG |  |  |
|  | ＜ 7.0 mmol/L (n = 43) | 53.5% (23/43) | 0.498 |
|  | ≥ 7.0 mmol/L (n = 89) | 47.2% (42/89) |  |
|  | Hypertension |  |  |
|  | Yes (n = 82) | 57.3% (47/82) | 0.206 |
|  | No (n = 50) | 46.0% (23/50) |  |
|  | Hyperlipemia |  |  |
|  | Yes (n = 102) | 54.9% (56/102) | 0.427 |
|  | No (n = 30) | 46.7% (14/30) |  |
|  | Cardiovascular and cerebrovascular diseases |  |  |
|  | Yes (n = 74) | 51.4% (38/74) | 0.662 |
|  | No (n = 58) | 55.2% (32/58) |  |
|  | Insulin treatment |  |  |
|  | Yes (n = 32) | 50.0% (16/32) | 0.693 |
|  | No (n = 100) | 54.0% (54/100) |  |
| Neutralizing antibody (B.A.4/5) | HbA1C |  |  |
|  | ＜ 7.0% (n = 58) | 12.1% (7/58) | 0.177 |
|  | ≥ 7.0% (n = 73) | 5.5% (4/73) |  |
|  | FPG |  |  |
|  | ＜ 7.0 mmol/L (n = 43) | 7.0% (3/43) | 0.695 |
|  | ≥ 7.0 mmol/L (n = 89) | 9.0% (8/89) |  |
|  | Hypertension |  |  |
|  | Yes (n = 82) | 8.5% (7/82) | 0.914 |
|  | No (n = 50) | 8.0% (4/50) |  |
|  | Hyperlipemia |  |  |
|  | Yes (n = 102) | 8.8% (9/102) | 0.707 |
|  | No (n = 30) | 6.7% (2/30) |  |
|  | Cardiovascular and cerebrovascular diseases |  |  |
|  | Yes (n = 74) | 8.1% (6/74) | 0.916 |
|  | No (n = 58) | 8.6% (5/58) |  |
|  | Insulin treatment |  |  |
|  | Yes (n = 32) | 0 (0/32) | 0.065 |
|  | No (n = 100) | 11.0% (11/100) |  |

Abbreviations: BMI, body mass index; FPG, fasting plasma glucose; HbA1c, hemoglobin A1c.
